# Increased BOLD Signals in dlPFC Is Associated With Stronger Self-Control in Food-Related Decision-Making

**DOI:** 10.3389/fpsyt.2018.00689

**Published:** 2018-12-20

**Authors:** Fuguo Chen, Qinghua He, Yan Han, Yunfan Zhang, Xiao Gao

**Affiliations:** ^1^Key Laboratory of Cognition and Personality (Ministry of Education), Southwest University, Chongqing, China; ^2^Faculty of Psychology, Southwest University, Chongqing, China; ^3^CAS Key Laboratory of Mental Health, Institute of Psychology, Beijing, China

**Keywords:** self-control, food choice, fMRI, dlPFC, decision-making

## Abstract

Self-control is the ability to comply with a request, to postpone acting upon a desire object or goal, and to generate socially approved behavior in the absence of external monitors. Overeating is actually the failure in self-control while feeding. However, little is known about the brain function that allows individuals to consciously control their behavior in the context of food choice. To address this issue, we used functional MRI to measure brain activity among undergraduate young females. Forty-one undergraduate female students participated in the current study. Subjects underwent the food rating task, during which they rated each food item according to their subjective perception of its taste (from Dislike it very much to Like it very much), its long term effect on health (from very unhealthy to very healthy) and decision strength to eat it (from Strong no to Strong yes). Behavioral results indicate the positive correlation between taste rating and its corresponding decision strength to eat, no matter the food is high caloric or low. Moreover, health ratings of high caloric food was negatively correlated with DEBQ-emotional eating, and taste ratings of high caloric food was positively correlated with DEBQ-external eating. Whole brain analysis of fMRI data indicates that BOLD responses in dlPFC were positively correlated with successful self-control; BOLD responses in midcingulate cortex were positively correlated with failed self-control. This study provided direct evidence that dlPFC was involved in self-control in food-related choice.

## Introduction

We are living in an environment which promotes over-consumption of palatably high-energy food, and the obesity epidemic shows no signs of abating (1). Individual differences in food reward sensitivity is responsible for overeating (2, 3). Sensitivity to food reward, insensitivity to internal state, and/or defects in impulsive control were found to predict overeating and a preference for foods high in fat and sugar. Moreover, the above these factors would, in turn, predict higher body mass index (BMI). Sensitivity to food reward works as the hot system which drives a person to food intake; impulsive control, on the contrary, works as the cool system which could restrains feed behavior. Numerous studies have focused on the hot system (2, 4, 5), whereas studies referring to cool mechanism in the brain is relatively scarce.

Self-control has been variously defined as the ability to comply with a request, to initiate and cease activities according to situational demands, to modulate the intensity, frequency, and duration of verbal and motor acts in social and educational settings, to postpone acting upon a desire object or goal, and to generate socially approved behavior in the absence of external monitors (6). Self-control is also defined as an umbrella construct that bridges concepts and measurements from different disciplines, such as impulsivity, conscientiousness, self-regulation, delay of gratification, inattention-hyperactivity, executive function, will power and inter temporal choice (7). Some scholars take self-control and self-regulation as the same concept. In general, self-regulation is the broader term, encompassing both conscious and unconscious processes and sometimes referring to all behavior guided by goals or standards, whereas self-control refers more narrowly to conscious efforts to alter behavior, especially restraining impulses and resisting temptations (8).

Overeating is actually the failure in self-control while feeding. High-sugar/high-fat food induce appetite-food reward, which drives food intake. Food reward is so strong that defect in self-control will, apparently, lead to overeating. Neuroimaging studies, using positron emission tomography (PET) and magnetic resonance imaging (MRI), which focused on food intake, yield valuable insights into the neurobiology underlying variation in regulation of food intake in human (9). Regulation of food intake is based on two major interacting systems: homeostatic needs affect the behavior mediated by gastrointestinal hormones and hypothalamic integration (integration of gastrointestinal hormone and hypothalamus function); the pleasure from food intake can provide reinforcement beyond homeostatic value and lead to overindulgence in high-caloric food (10). The hedonic component of feeding behavior is suggested to be mediated by reward-related cortical and sub-cortical systems, that is, the ventral striatum, the ventral tegmental area and the orbitfrontal cortex (OFC). However, little is known about the brain function that allows individuals to consciously control their behavior in the context of food choice.

Neuroimaging studies focusing on self-control found that cognitive control is the highest level of cognitive activity, and prefrontal lobe plays as the central role in cognitive control system (11–13). Difference sub-region of PFC perform its own functions: the ventromedial prefrontal cortex (vmPFC) is responsible for reward assessment and goal orientation; the dorsolateral prefrontal cortex (DLPFC) is in charge of process of self-control; the medial orbitofrontal cortex (mOFC), via the lateral prefrontal cortex (lPFC), dedicates to represent subjective reward values at the time of choice (14–16). Other brain areas like insula and its relying operculum also participate in self-regulation of cognitive control function s (17).

Contrary to the activity of self-control system, the brain reward system, containing vmPFC, lPFC, OFC, striatum, insula, anterior cingulum gyrus cortex, hippocampus, amygdale, and midbrain structures, encodes the subjective value of rewards and the subsequent impulse system, and it consistent with a role for this neuronal network in general hedonic representation (18, 19). Reward activity in lPFC and vmPFC represent characteristics and intense of anticipation to reward (11, 20), and striatum is responsible for coding “liking” and “wanting” qualities of food (21). Amygdala, with projections to nucleus accumbens to trigger motivitioanal behaviors, functions as emotional integration, reward process and feeding regulation (22). Hippocampus, the central part of memory, conserve the pleasure feeling and emotional reaction, and then transport these information to dosal stratum and cerebellum, regulating the feeding or avoiding behavior (23).

Along with previous literature, there is, apparently, an antagonistic effect between the self-control system represented by prefrontal area and the impulsive system represented by striatum. In a particular situation, intense activities in the prefrontal system indicate much weaker activities in the stratum system, and vice versa. It can be summarized that the balance between these two brain systems are vital to regulate feed behavior. Overeating and obesity will happen very likely if the balance is broken. We speculate that delicious high-fat food, which is difficult to resist and reject, may disturb the balance between the self-control system and the impulsive system. Previous studies mainly focused on the activities in stratum system while watching food pictures or the anticipating to intake food. Evidence in prefrontal control mechanism of feeding behavior is scarce. Hence, the current study, based on the activities of the prefrontal system while watching various food pictures, aimed to investigate the intrinsic mechanism underlying the self-control function in food-related decision-making. We employed three food rating tasks in the current study. In these tasks, subjects were required to indicate their decision intensity to consume the food items based on balancing the immediate pleasure from their taste and its long-term effect on health. We categorize subjects as high self-control group or low self-control group according to their score in food rating tasks. We hypothesized that: (1) increased dlPFC function would associate with self-control; (2) food picture would activate numbers of related areas, such as somatosensory cortex (postcentral gyrus), visual cortex (superior parietal lobule, cuneus), primary taste cortex (insular), rewarding areas (striatum, OFC); (3) activity differences would be observed between food-acceptation and food-rejection.

## Methods

### Subjects

Forty-three subjects participated in the current experiment [all females; mean age = 20.47 years, S.D. = 1.75, age range = 18–25 years; mean BMI = 23.05, S.D. = 4.44, BMI range = 15.56–29.32]. The demographic information of subjects was presented in Table 1. Due to excessive head movement during scanning, four subjects (who exceeded a predetermined limit of 2 mm in any direction) were excluded from the sample. Findings from the resulting sample of 41 were reported. All subjects were right-handed nonsmokers, with no reported past/current neurological or psychiatric illness, normal or corrected-to-normal vision and normal color vision as assessed by basic color tests. None of them took medications. We did not impose a BMI upper-limit or lower-limit. Subjects were included as long as they felt comfortable while inserted in the fMRI scanner. All subjects provided the date of their last period to ensure that they were not scanned during menstruation.

**Table 1:** Demographic information of the subjects.

**Variables**	**Whole sample(*****N*** **=** **41)**		
	**Range**	**M**	**SD**
Age	18–25	20.56	1.73
BMI	15.56–28.94	22.85	4.43
**DEBQ**			
DEBQ-R	1.20–4.00	2.88	0.75
DEBQ-EM	1.15–3.77	2.4	0.67
DEBQ-EX	2.30–4.30	3.21	0.49
**BEHAVIORAL RATINGS**			
Taste ratings	2.32–3.33	2.77	0.21
Taste ratings	2.27–3.15	2.75	0.21
Decision ratings	2.19–3.17	2.71	0.19

*DEBQ-R, Restraint subscale; DEBQ-EM, Emotional Eating subscale; DEBQ-EX, External Eating subscale; HC, High caloric food; LC, Low caloric food*.

### Measures

#### Demographics

Subjects completed a demographics questionnaire, including age, current and historic medications, and phase of menstrual cycle.

#### Hunger Ratings

Subjects rated current feelings of hunger on a 5-point Likert scale, ranging from 0 (“*not at all hungry*”) to 4 (“*very hungry*”).

Dutch Eating Behavior Questionnaire [DEBQ; (24)] was used to assess subjects' eating behavior. It consisted of three subscales, including the Emotional Eating subscale (DEBQ-EM; 13 items; e.g., the degree to which eating is prompted by emotional states like tension and worry rather than by hunger), the External Eating subscale (DEBQ-EX; 10 items; e.g., the degree to which one tends to overeat if food looks and smells good), and the Restraint subscale (DEBQ-R; 10 items; e.g., the extent to which the individuals restrain food intake). Impulsivity in eating behaviors could be reflected by the DEBQ-EM and DEBQ-EX. DEBQ has 33 items in total, and each item was measured on a 5-point Likert scale. It has good reliability and validity. The Cronbach's α of each subscale in the current sample was 0.95, 0.81, and 0.95, respectively.

### Stimuli

One hundred and seventy different food items with 85 picturing high-caloric (HC) palatable food (e.g., fried chicken, hot dog, ice cream, etc.) and 85 picturing low-caloric (LC) food (e.g., fruits, vegetables, etc.) were used in the current study. All of the stimuli were adopted from Chinese Food Picture Database (25). The food pictures were presented to the subjects using color pictures (72 dpi). Stimulus presentation and response recording was controlled by E-prime 2.0.

#### Food Rating and Decision-Making Task

The food rating and decision-making task was similar with the task used in previous study (26). The task had three parts. Subjects first rated all 80 high-caloric and 80 low-caloric food items for both their taste and their long term effect on health in two separate blocks (a taste-rating block and a health-rating block). All ratings were made using a four-point scale that was shown on the screen below each item. The taste ratings were made on a 4-Likert scale from 1 = *Dislike it very much*, 2 = *Dislike it*, 3 = *Like it*, to 4 = *Like it very much*. And all health ratings were made on a 4-Likert scale from 1 = *Very unhealthy*, 2 = *Unhealthy*, 3 = *Healthy*, to 4 = *Very healthy*. Subjects were instructed to rate the taste without regard for its healthiness before the taste-rating block. Similarly, before the health-rating block they were instructed to rate the healthiness of each food item without regard for its taste. After the two rating blocks, subjects were presented with another food picture gallery including 10 food items (5 high-caloric and 5 low-caloric items), and they were asked to choose one food item as a reference item, which was relatively neutral on their taste and health perception.

In decision phase, all subjects were presented with the 160 food items again and were instructed that on each trial they would have to choose between eating the food item shown in that trial and the reference food item. Subjects were told to express the strength of their preferences using a four-point scale: 1 = *Strong No* (choose reference food), 2 = *No* (choose reference food), 3 = *Yes* (choose shown food), 4 = *Strong Yes* (choose shown food) (26).

The taste-rating task and decision-making task was done in the fMRI scanner and the health-rating task was done out of the scanner.

### Procedure

Following approval from the Human Research Ethics Committee at School of Psychology, Southwest University, subjects were recruited via on-campus advertisements. Subsequently, 43 female undergraduate students engaged in the current study. All of the subjects took part in an intake session and one fMRI scanning session. Two sessions were conducted on separate days. Body weight and height was measured during the intake session. BMI was calculated as weight (in kilograms) divided by the squared height (in meters) of the subject (BMI = kg/m^2^). Specifically, after the removal of shoes and coats, height was measured to the nearest millimeter using a stadiometer and weight was assessed to the nearest 0.1 kg using a digital scale. During the intake session, subjects signed the consent inform after reading a general overview of the study. Anthropometric measurements were then taken.

On the day of the fMRI scan, subjects were instructed to refrain from eating or drinking, with the exception of water, within 12 h before their session. Fasting status was confirmed by self-report questionnaires upon their arrival. Then subjects were introduced with the taste and health rating task, as well as the decision-making task. After that, subjects did the health rating task out of the fMRI scanner, and chose the reference food item. Then, subjects were taken into the scanner bore, and they did the hunger rating right before the structural image acquisition. After the rating, T1-weighted structural scans and the resting state fMRI (rs-fMRI) run was conducted. Then subjects completed the food taste rating runs and decision-making runs in the scanner, sequentially. Only the results from the decision-making task were reported here and the resting state fMRI data were reported in another study (27). Each subject was paid 140 Yuan as compensation for their participating after the fMRI scanning session.

### fMRI Data Acquisition

fMRI data were acquired using a 3-T Siemens Trio scanner in the SWU Imaging Center for Brain Research. Foam pads were used to reduce head movements and scanner noise. Scans were performed by an echo-planar imaging (EPI) sequence with the following scan parameters: repetition time = 2,000 ms, echo time = 30 ms, flip angle = 90°, field of view = 192 × 192 mm^2^, acquisition matrix = 64 × 64, in-plane resolution = 3 × 3mm^2^, 32 interleaved 3-mm-thick slices, inter-slice skip = 0.99 mm. Two volumes were discarded before the beginning of data collection in each run to allow for equilibration of the magnetic field.

### Data Preprocessing

Neuroimaging data were preprocessed using the SPM12 software (Statistical Parametric Mapping, Wellcome Department of Imaging Neuroscience, London, United Kingdom) on the Matlab platform. For each subject, the first 10 volumes were discarded to account for signal equilibrium and subjects' adaptation to their immediate environment. Then, the fMRI images were corrected for the acquisition delay between slices and for the head motion. Two subjects were excluded because their head motion exceeded 2 mm in translation or 2° in rotation. Then, anatomical and functional images were normalized to the standard MNI template brain implemented in SPM12, resulting in voxel sizes of 1 and 3 mm^3^, respectively. Functional time-series data were then detrended.

### Statistical Analysis

#### Behavioral Data

Firstly, descriptive analysis was conducted with the demographic variables, DEBQ and behavioral ratings of the taste, health and decision making. Then, correlation analysis was conducted between decision strength of HC food or LC food, DEBQ-EM, and DEBQ-EX, respectively. We expected that decision strength-HC would positively correlate with DEBQ-EM and DEBQ-EX, while decision strength-LC would show no such correlation with these variables. According to behavior scores in both the food rating task and decision making task, we divided subjects into three groups: success in self-control (SSC), failure in self-control (FSC) and no self-control (NSC). SSC means rejection to like/healthy food and acceptation to dislike/healthy food; FSC means rejection to unlike/healthy food and acceptation to like/unhealthy food; NSC means rejection to unlike/unhealthy food and acceptation to like/healthy food.

#### fMRI Data

##### Whole brain analyses

Analysis was performed with SPM12. Individual level whole brain general linear models (GLMs) and SPM12's standard hemodynamic response function was estimated in three steps. Firstly, we estimated the model separately for each individual. Three events were defined: (1) success in self-control (SSC) including choosing healthy-disliked food and rejecting unhealthy-liked food; (2) failure in self-control (FSC) including rejecting healthy-disliked food and choosing unhealthy-liked food; and (3) no self-control (NSC) including trials with healthy-liked food and unhealthy-disliked food. Secondly, we calculated contrast statistics at the individual level. Two main contrasts were specified for subject-level analysis: (1) SSC vs. FSC and (2) FSC vs. SSC. Thirdly, a general linear model was used to generate the statistical parametric maps for the second-level analysis, while BMI was introduced as a covariate variable in the analysis in the original manuscript. We expected that increased dlPFC function would associate with self-control. Main effects were considered significant using a whole-brain family wise error (FWE) of *p* < 0.05 and a minimum cluster size of 5 voxels.

## Results

### Behavioral Results

The results of descriptive statistical analysis on three rating tasks were shown in Table 1, and the inter-correlation between variables of interest was presented in Table 2. Results show that taste-likeness for low caloric food negatively correlated with DEBQ-EM (*r* = −0.32, *p* = 0.05); taste-likeness for high caloric food positively correlated with DEBQ-EX (*r* = 0.35, *p* = 0.05); there is positive correlation between food liking and its corresponding eating choice, no matter the food is high caloric or low; health assessment of high caloric food negatively correlated with DEBQ-EM (*r* = −0.32, *p* = 0.05); likeness assessment of high caloric food positively correlated with DEBQ-EX (*r* = 0.33, *p* = 0.05).

**Table 2:** Inter-correlation matrix between variables.

**Variables**	**1**	**2**	**3**	**4**	**5**	**6**	**7**	**8**
1 Taste-HC	1							
2 Taste-LC	−0.213							
3 Health-HC	0.390^*^	−0.056						
4 Health-LC	0.105	0.564^**^	0.111					
5 Decison-HC	0.748^**^	−0.119	0.510^**^	0.135				
6 Decison-LC	−0.262	0.846^**^	−0.185	0.433^**^	−0.309^*^			
7 DEBQ-R	0.032	−0.079	−0.105	0.039	−0.026	0.052		
8 DEBQ-EM	0.029	0.21	−0.322^*^	0.013	−0.106	0.26	0.089	
9 DEBQ-EX	0.331^*^	−0.049	−0.141	0.028	0.094	−0.027	0.092	0.525^**^

*HC, High caloric food; LC, Low caloric food; DEBQ-R, Restraint subscale; DEBQ-EM, Emotional Eating subscale; DEBQ-EX, External Eating subscale*.

* *p < 0.05 two tailed*.

** *p < 0.01 two tailed*.

### Brain Image Results

**Figure 1:**
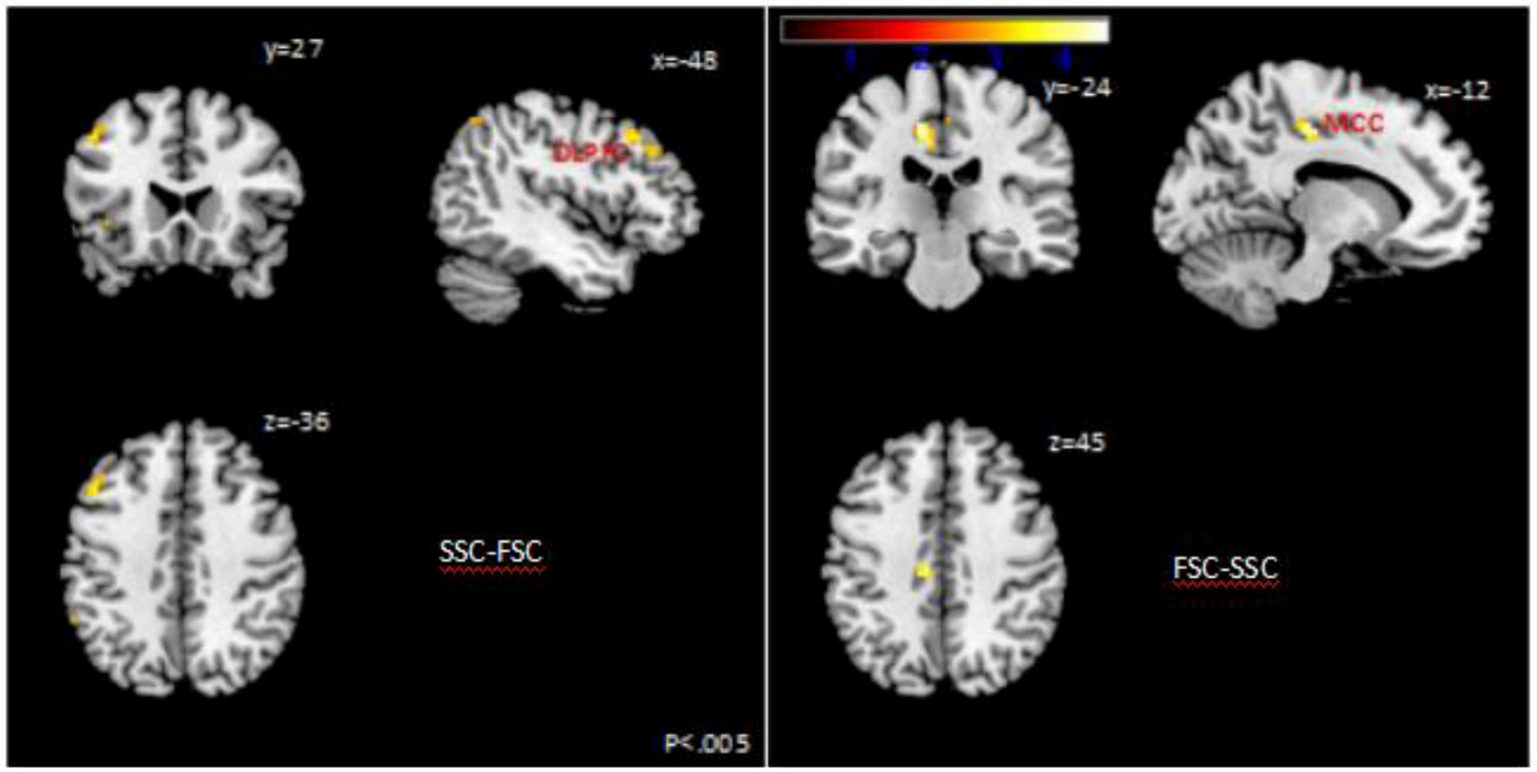
Activation map of Successful self-control (SSC-FSC) vs. Failed self-control (FSC).

We estimated a general linear model of brain responses in which activity during the entire evaluation period was modulated by self-control. Whole brain analysis showed that SSC vs. FSC significantly activated BOLD responses in DLPFC, whereas FSC vs. SSC significantly activated BOLD responses in midcingulate cortex (MCC) (Table 3 and Figure 1).

**Table 3:** Brain regions showing significant correlation between the activity area and self-control.

**Brain region**	**MNI coordinates**			***Z***	***p***
	***X***	***Y***	***Z***		
SSC-FSC					
DLPFC	−48	27	36	3.47	0.026
FSC-SSC					
MCC	−12	−24	45	4.35	0.008

*SSC, Successful self-control; FSC, Failed self-control*.

### Correlation With DEBQ

Correlation analysis was performed with the SSC vs. FSC and FSC vs. SSC maps against individual's scores of three DEBQ subscales. However, no significant finding was obtained.

**Figure 2:**
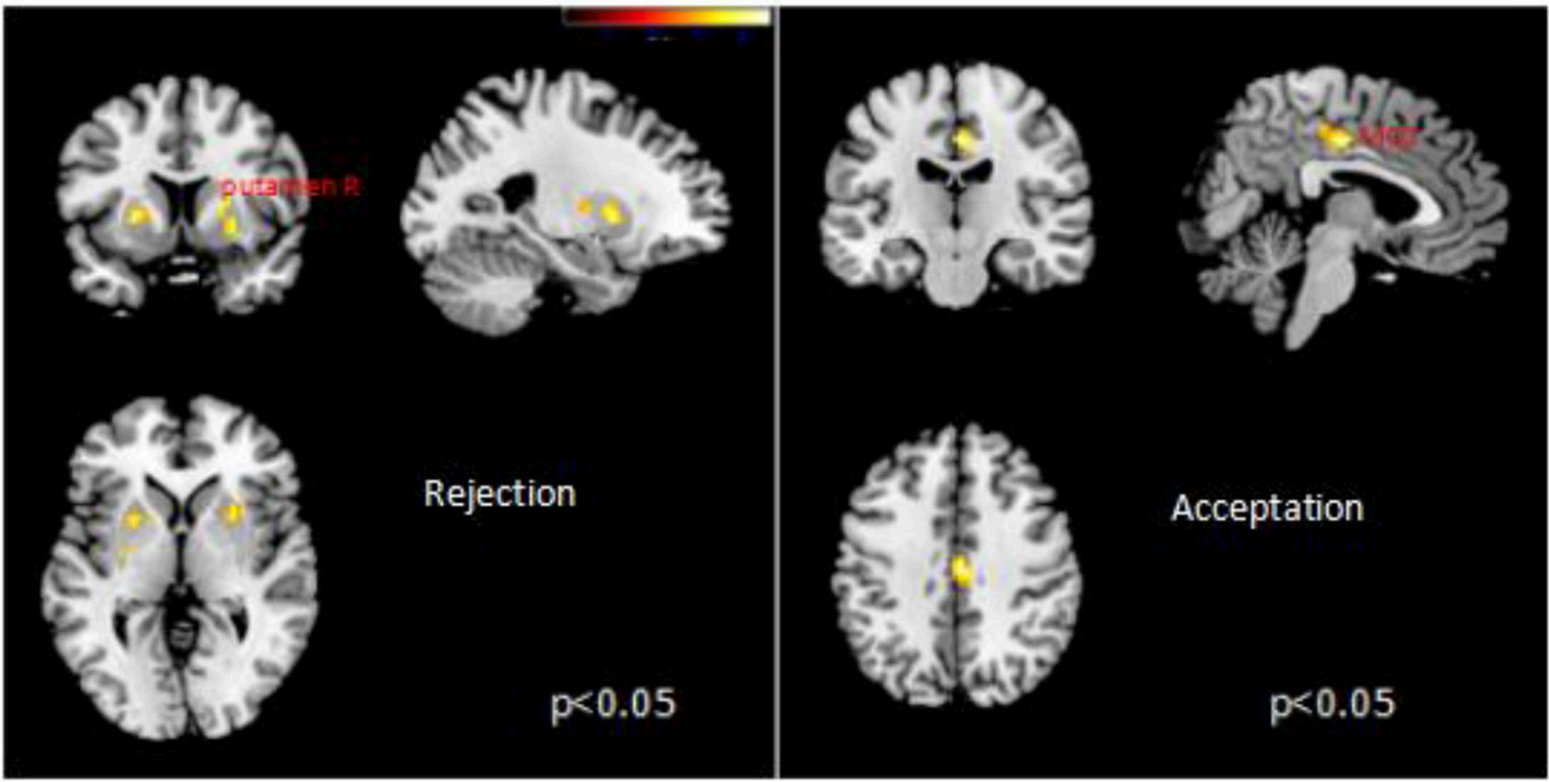
Correlation between SSC-R vs. FSC-R **(left)**, SSC-C vs. FSC-C **(right)** and DEBQ-R.

Then, we divided SSC into two events at individual level: (1) choosing healthy-disliked food (SSC-C) and (2) rejecting unhealthy-liked food (SSC-R). Meanwhile, FSC were divided into two events at individual level: (1) choosing unhealthy-liked food (FSC-C) and (2) rejecting healthy-disliked food (FSC-R). Four main contrasts were specified for subject-level analysis: (1) SSC-R vs. FSC-R, (2) FSC-R vs. SSC-R, (3) SSC-C vs. FSC-C and (4) FSC-C vs. SSC-C. Correlational analysis were performed with the four contrast maps against individual's scores of three DEBQ subscales. Results showed that BOLD responses in bilateral putamen were positively correlated with DEBQ-R on SSC-R vs. FSC-R contrast. Meanwhile, BOLD responses in left MCC were positively correlated with DEBQ-R on FSC-C vs. SSC-C (Table 4 and Figure 2).

**Table 4:** Brain regions showing significant correlation between the activity area and DEBQ.

**Brain region**	**MNI coordinates**			***Z***	***p***
	***X***	***Y***	***Z***		
SSC-R vs. FSC-R					
putamen R	27	6	−6	4.05	0.014
putamen L	−21	9	3	3.86	0.028
SSC-C vs. FSC-C					
MCC	3	−18	42	4.21	0.015
FSC-C vs. SSC-C					
MCC	−12	−24	45	4.35	0.008

*SSC, Successful self-control; FSC, Failed self-control; MCC, Midcingulate cortex*.

## Discussion

Based on the reinforcement learning hypothesis of self-control and food decision making, this study provides an approach to characterize the association between self-control on food-related choice.

As expected, self-control, which was indexed by the food choice between two food items, was positively correlated with BOLD responses in dlPFC. This is direct evidence showing the importance of dlPFC on self-control. Meanwhile, BOLD responses in MCC were positively correlated with unsuccessful self-control. These findings were in line with previous observations of the positive association between dlPFC with inhibition to energy intake (28), or smokers (29, 30). Dorsal lateral PFC also functions as the translation mechanism, which reinforces self-power, motivating one's goal-directed behavior in the long run (31, 32). DLPFC also projects to other brain areas, promoting or inhibiting various neuro-functions, such as executive control and emotion regulation n (33, 34). Subjects with damaged dlPFC had difficulty in focusing on cognitive task, implying a defect in self-control (35).

Under the “rejection” conditions, BOLD responses in bilateral putamen were positively correlated with DEBQ-R, no matter it's successful or unsuccessful self-control; Under the “acceptation” conditions, responses in MCC, unilaterally, were positively correlated with DEBQ-R, no matter it's successful or unsuccessful self-control. Although activities can be observed under both condition, we speculate there may exist difference between them, which implying different neuromechanisms between “accepting” food and “rejecting” food. Cingulate gyrus is crucial structure functioning in the regulation system (36). Neuroimaging studies referred to food pictures found that cingulate gyrus was stably activated during the attention to food stimulus (28, 37). In our daily life, while choosing food, we have to face the conflict which was caused by flavor of food and its healthy meaning to our bodies, and self-control help to regulate feeding behavior. In the current study, we defined successful self-control as rejection to like/healthy food or acceptation to dislike/healthy food; we defined unsuccessful self-control as rejection to unlike/healthy food or acceptation to like/unhealthy food. Based on the definition, subjects who have weaker self-control may usually choose food with better flavor, ignoring its impact on health. Functional MRI results in the study presented evidence implying that cingulate gyrus had stronger BOLD reaction when subjects focused on the flavor of food.

Interestingly, fMRI results in the current study also confirmed that DEBQ scores had positive correlation with BOLD responses in putamen, bilaterally, no matter self-control is successful or unsuccessful. Studies concerning restrained eating verify that high score of DEBQ reveals stronger cognitive control while in feeding environment, which, consequently, lead to restricted eating behavior (10). Besides putamen, dorsal striatum and caudatum may function to regulate restricted eating (10, 38). Studies concerning the old reveal similar conclusion, that putamen, caudatum and dlPFC collaborate with each, functioning as the self-control system to regulate delay of discounting and delay of gratification (39). Inversely, defect collaboration among these areas may lead to the reduction of resisting food temptation (40). What's more interesting is that, in the successful self-control trails, “acceptation” and “rejection” respectively activated different areas. “Acceptation” decision activated MCC and “rejection” the putamen. This underlies that these two decision-making behaviors may associated with different mechanism, despite they were both “successful” self-control reaction. Successful self-control owes to two ways: one is to resist the current temptation; the other is to think a lot of the future reward. From this perspective, successful self-control equals to the ability to choose health food, which is in accordance with long-term goals and succeeding in losing weight requires both behaviors (41).

## Ethics Statement

This study was carried out in accordance with the recommendations of The Regulations of Ethical Reviews of Biomedical Research Involving Human, Ministry of Health, China. The protocol was approved by the Ethic Committee of Faculty of Psychology, Southwest University. All subjects gave written informed consent in accordance with the Declaration of Helsinki.

## Author Contributions

FC: wrote this manuscript. YZ and YH: analyzed the data. QH: edited the manuscript and did the proof reading. XG: designed this study and got the funding.

### Conflict of Interest Statement

The authors declare that the research was conducted in the absence of any commercial or financial relationships that could be construed as a potential conflict of interest.
